# Forkhead Protein FoxO1 Acts as a Repressor to Inhibit Cell Differentiation in Human Fetal Pancreatic Progenitor Cells

**DOI:** 10.1155/2017/6726901

**Published:** 2017-02-28

**Authors:** Zongzhe Jiang, Jingjing Tian, Wenjian Zhang, Hao Yan, Liping Liu, Zhenhe Huang, Jinning Lou, Xiaosong Ma

**Affiliations:** ^1^Shenzhen University Diabetes Institute, Shenzhen University, Shenzhen 518060, China; ^2^Institute of Clinical Medical Sciences, China-Japan Friendship Hospital, Beijing 100029, China; ^3^Shenzhen Hightide Biopharmaceutical Ltd., Shenzhen 518000, China; ^4^Department of Aging Medicine, The Sixth Hospital of Shenzhen Municipality, Shenzhen 518060, China

## Abstract

Our colleagues have reported previously that human pancreatic progenitor cells can readily differentiate into insulin-containing cells. Particularly, transplantation of these cell clusters upon in vitro induction for 3-4 w partially restores hyperglycemia in diabetic nude mice. In this study, we used human fetal pancreatic progenitor cells to identify the forkhead protein FoxO1 as the key regulator for cell differentiation. Thus, induction of human fetal pancreatic progenitor cells for 1 week led to increase of the pancreatic *β* cell markers such as Ngn3, but decrease of stem cell markers including Oct4, Nanog, and CK19. Of note, FoxO1 knockdown or FoxO1 inhibitor significantly upregulated Ngn3 and insulin as well as the markers such as Glut2, Kir6.2, SUR1, and VDCC, which are designated for mature *β* cells. On the contrary, overexpression of FoxO1 suppressed the induction and reduced expression of these *β* cell markers. Taken together, these results suggest that FoxO1 may act as a repressor to inhibit cell differentiation in human fetal pancreatic progenitor cells.

## 1. Introduction

Decrease of *β* cell mass plays a crucial role in development of type 2 diabetes mellitus. Islet transplantation is a promising strategy to reestablish the *β* cell mass; however, its usage is limited by the shortage of available islets [1]. Human fetal pancreatic stem cells have been found as a good source of insulin producing cells, given its capability of readily self-renewal and differentiating into insulin producing cells in vitro by differentiation at conditions resembling those of physiological environments [2]. Our colleagues have reported previously that these differentiated cell clusters generated from human fetal pancreatic progenitor cells exhibited more insulin contents and improved secretary capability and glucose response [3]. Transplantation of these cell clusters normalized hyperglycemia in diabetic nude mice [3]. Nevertheless, the key molecular in controlling differentiation of the human fetal pancreatic progenitor cells is still unknown.

It has been found that the forkhead transcription factor FoxO1 is a prominent mediator in controlling pancreatic *β* cell mass [4]. FoxO1 is most abundant isoform among FOXO class members in the adult pancreas and preferentially expressed in pancreatic *β* cells, where it plays an essential role in *β* cell growth and proliferation [5, 6]. During mouse pancreatic organogenesis, FoxO1 is found in the pancreatic epithelium between e9.5 and 14.5 [7] and is implicated in pancreatic organogenesis [7]. Previous studies revealed that FoxO1 ablation in mice resulted in increase of juxtaductal *β* cells [8] and insulin-positive cells generated from the gut epithelial cells [9]. Moreover, FoxO1 knockdown rescued the diabetic phenotype in insulin-resistant mice [10], whereas constitutive activation of FoxO1 caused hyperglyceridemia and impaired insulin secretion [11]. However, little is known of its role in regulation of *β* cell development in the human fetal pancreas. In this study, we used human fetal pancreatic progenitor cells to identify the role of FoxO1 in cell differentiation.

## 2. Materials and Methods

### 2.1. Culture of Human Fetal Pancreatic Progenitor Cells

The present study was approved by the Clinical Research Ethics Committee of both Shenzhen University and China-Japan Friendship Hospital and conducted according to the principles of the Declaration of Helsinki. The human fetal pancreatic progenitor cells used for expansion were cultured in a 37°C, 5% CO_2_ incubator in DMEM/F12 medium containing 5% fetal bovine serum, 40 *μ*g/L leukemia inhibitor factor (LIF), 10 *μ*g/L basic fibroblast growth factor (bFGF), 10 *μ*g/L epidermal growth factor (EGF), 10^5^ U/L penicillin, and 100 mg/L streptomycin.

### 2.2. Induced Differentiation of Human Pancreatic Progenitor Cells

The expansion induction of human fetal pancreatic progenitor cells was as described previously [3]. Thus, human pancreatic progenitor cells at the 10th gestational week were induced in M199 medium containing 15% fetal bovine serum, 10 mmol/L nicotinamide, 30 ng/mL all-trans retinoic acid, and 42 ng/ml glucagon-like peptide-1 for 1 week. The medium was replaced every three days.

### 2.3. RNA Isolation and Quantitative Real-Time PCR Analysis

Total RNA from induced hFPPCs was extracted using RNAiso Plus (TaKaRa Biotechnology, Dalian, China). Single-stranded cDNAs were generated with Bestar™ qPCR RT Kit (DBI Bioscience, Shanghai, China). Real-time PCR was conducted by using Bestar qPCR Mastermix SYBR green (DBI Bioscience, Shanghai, China) in ABI prism 7500 Sequence Detection System. Analysis of relative gene expression was measured by quantitative real-time PCR and the 2^−ΔΔCT^ Method. The pancreatic stem cell markers (Oct4, Nanog and CK 19), as well as endocrine and *β* cell markers (Ngn3, insulin, GLUT2, Kir6.2, SUR1, and VDCC), were evaluated during differentiation. The mRNA levels of tested markers were normalized to GAPDH.

### 2.4. Western Blotting

Cell pellets were incubated in RIPA lysis buffer (Beyotime, Nantong, China) supplemented with 1 mM protease inhibitor cocktail (CALBIOCHEM, USA) for 30 minutes on ice, followed by centrifugation at 12,000 rpm for 10 minutes at 4°C. Cell lysates were resolved using SDS-PAGE gels and transferred onto a polyvinylidene difluoride (PVDF) membrane by electrophoresis. The membranes were immunoblotted with the monoclonal rabbit anti-FoxO1 (1 : 1000, Cell Signaling, Danvers, MA, USA); the monoclonal mouse anti-*β*-actin (1 : 2000, Santa Cruz Biotechnology, Inc., Santa Cruz, CA, USA), followed by incubation with a goat anti-rabbit secondary antibody (1 : 3000, Santa Cruz Biotechnology, Inc., Santa Cruz, CA, USA) at room temperature for 2 h. Immunoreactive bands were revealed by enhanced chemiluminescence (SuperSignal® West Pico Chemiluminescent Substrate kits, Thermo Scientific) and visualized by the KODAK Image Station 4000MM PRO imaging system and software. Band intensities were quantified by scanning densitometry (Gel-Doc2000, Bio-Rad), analyzed with Quantity One™ (Bio-Rad), and normalized against the level of *β*-actin.

### 2.5. RNAi Transfection

Transfection of FoxO1-siRNA and control siRNA was according to the typical RNAiMAX transfection procedure. Briefly, siRNA was diluted with Opti-MEM Medium into 0.2 *μ*m/L and mixed with diluted Lipofectamine RNAiMAX Reagent. The mixture was incubated for 5 minutes at room temperature and the siRNA-lipid complex was added into cells afterwards.

### 2.6. Insulin Content Measurement

Human fetal pancreatic progenitor cells transfected with siRNAs were collected and lysed for protein extraction. 25 *μ*l supernatant of sample lysate was used for insulin measurement with human insulin ELISA kits (ALPCO Diagnostic, Salem, NH, USA).

### 2.7. Immunofluorescent Staining

For immunofluorescent staining, human fetal pancreatic progenitor cells transfected with siRNAs were fixed in 4% paraformaldehyde in PBS for 30 min at room temperature. Then, they were transferred to membrane permeabilization solution (0.3% Triton X-100) for 20 min and blocking buffer (1% BSA-supplemented PBS) for 1 h. At last, cells were incubated overnight at 4°C with antibodies in appropriate dilutions.

### 2.8. Retroviral Infection

pMX-puro-FoxO1-AAA and control vector for retroviral packaging were cotransfected with psi-2 helper plasmid into 293 T cells using the calcium chloride precipitation method. The generated recombinant virus was collected and transfected into hFPPCs, followed by selection in 2 *μ*g/ml puromycin (Sigma) for 4 days.

### 2.9. Statistical Analyses

Data are presented as mean ± SEM for the indicated number of experiments (*n*). Statistical significance was evaluated using the independent *t*-test. Data were considered significant when *p* < 0.05.

## 3. Results

### 3.1. Induction of Human Fetal Pancreatic Progenitor Cells

**Figure 1:**
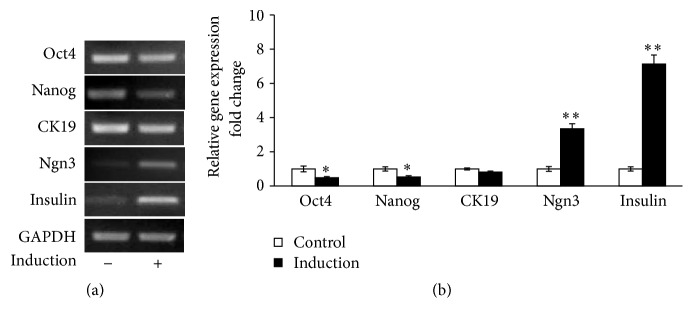
Induction of human pancreatic progenitor cells. (a) Human fetal pancreatic progenitor cells of 10 week were cultured and induced for 1 week. RT-PCR analysis was used to evaluate the markers for stem cell and pancreatic *β* cell in cells before and after 1-week induction. GADPH was used as internal control. (b) Values were expressed as percentage of mRNA expression in cells of induction before (empty bars) and 1-week induction (black bars). Data are means ± SEM of 4 independent experiments per each group. *∗* or *∗∗*, *p* < 0.05 or 0.01.

Human pancreatic progenitor cells derived from 10-week fetal pancreas were induced for differentiation for 7 days as described before [3]. We first examined the expression of the stem cell markers (Oct4 and Nanog) [12, 13], pancreatic ductal cell markers (CK19), pancreatic endocrine marker (Ngn3), and the *β* cell marker (insulin) in human pancreatic progenitor cells before and after 7-day induction. qRT-PCR analyses revealed that mRNA levels of Oct4, Nanog, and CK19 were decreased upon induction. By contrast, levels of Ngn3 and insulin designated for endocrine and pancreatic *β* cells were significantly increased (Figures 1(a) and 1(b)), which were consistent to the observations made in the same in vitro induction of the human fetal pancreatic progenitor cells [3].

### 3.2. Characterization of FoxO1 Expression in the Development of Human Fetal Pancreatic Progenitor Cells

**Figure 2:**
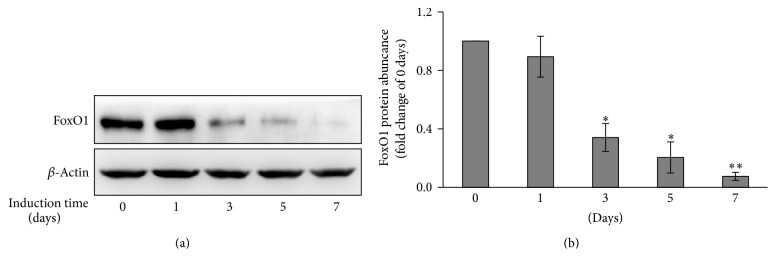
Reduced FoxO1 protein level during induction of human pancreatic progenitor cells. (a) Time course analysis of FoxO1 expression in human pancreatic progenitor cells after induction of differentiation for the time as indicated. Expression of FoxO1 protein was determined by Western blotting. *β*-Actin was used as loading control. (b) Data were normalized to the level of *β*-actin and expressed as fold change relative to control. Values are means ± SEM of 3 independent experiments per each group. *∗* or *∗∗*, *p* < 0.05 or 0.01.

The temporal profiles of FoxO1 were analyzed before and after 7-day induction of human fetal pancreatic progenitor cells by Western blotting. As shown in Figure 2(a), FoxO1 protein level decreased in a time-dependent manner during 7-day induction. Indeed, FoxO1 protein level at the 7th day was 7.5 ± 2.6% (*p* < 0.01) of that at control (before induction) (Figure 2(b)).

### 3.3. Knockdown of FoxO1 Promotes Cell Differentiation in Human Fetal Pancreatic Progenitor Cells

**Figure 3:**
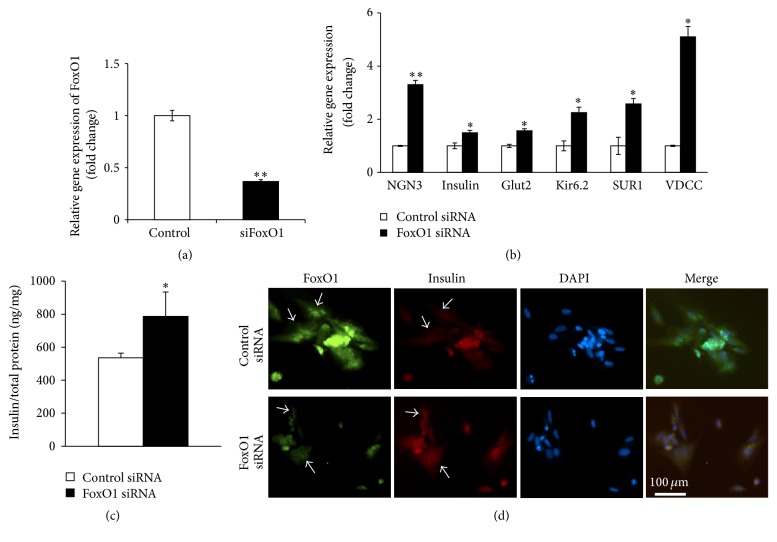
Knockdown of FoxO1 results in increased expression of *β* cell markers. (a) qRT-PCR analysis of FoxO1 mRNA levels in human pancreatic progenitor cells transfected with scramble or FoxO1 siRNA for 48 h. Data were normalized against the level of *β*-actin and expressed as fold change relative to scramble siRNA group. Values are means ± SEM of 4 independent experiments per each group. ^*∗∗*^*p* < 0.01. (b) qRT-PCR analysis of *β* cell markers in human pancreatic progenitor cells transfected with scramble or FoxO1 siRNA for 48 h. Data were expressed as fold change relative to scramble siRNA group and are means ± SEM of 4 to 5 independent experiments. *∗* or *∗∗*, *p* < 0.05 or 0.01. (c) Insulin content analysis by ELISA for human fetal progenitor cells, respectively, transfected with FoxO1 siRNA or control siRNA. Values were normalized to total protein content. ^*∗*^*p* < 0.05. (d) Immunohistochemistry with antibodies against FoxO1 (green, dilution of 1 : 100) and insulin (red, dilution of 1 : 100) in the human fetal progenitor cells, respectively, transfected with FoxO1 siRNA (under panels) or control siRNA (upper panels). Arrows indicate the FoxO1 knockdown cells where the insulin was also decreased. Bar = 100 um.

**Figure 4:**
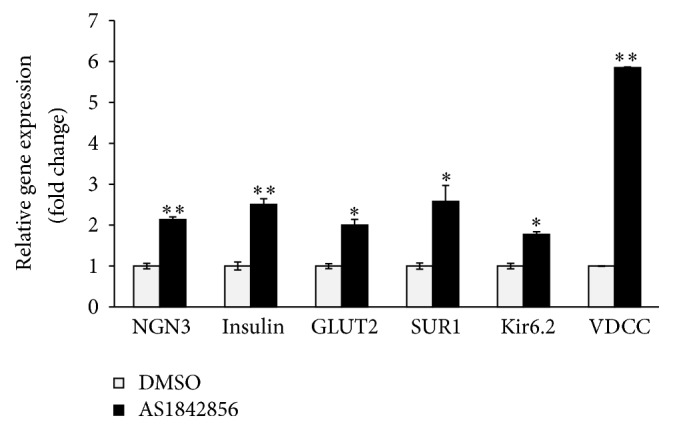
FoxO1 inhibitor increased mRNA expression of *β* cell markers. Human fetal pancreatic progenitor cells were treated with DMSO or AS1842856 for 3 days. qRT-PCR analysis of *β* cell markers. Data were expressed as fold change relative to DMSO group and are means ± SEM of 4 independent experiments per each group. *∗* or *∗∗*, *p* < 0.05 or 0.01.

We next determined the role of FoxO1 in the induction of human fetal pancreatic progenitor cells. The experiments were performed by transfection of FoxO1 siRNA in the human pancreatic progenitor cells for 24 h. RNAi transfection resulted in ~65% reduction of FoxO1 level in human fetal pancreatic progenitor cells (Figure 3(a)). This is accompanied by significant increase of Ngn3, insulin, Glut2, Kir6.2, SUR1, and VDCC, as compared to cells transfected with control siRNA (Figure 3(b)). Consistent with the findings by the qRT-PCR analysis, progenitor cells transfected with FoxO1 siRNA showed substantial increase in insulin content (Figure 3(c)), as well as insulin immunoreactivity (Figure 3(d)). This result was supported by the experiments of treatment of human fetal pancreatic progenitor cells with 0.1 *μ*M AS 1842856, the specific FoxO1 inhibitor [14] for 6 days. In this series of experiments, treatment of FoxO1 inhibitor resulted in ~2- to ~6-fold (*p* < 0.05 or 0.01) increase of mRNA levels for Ngn3, insulin, Glut2, SUR1, and VDCC, respectively (Figure 4).

### 3.4. Constitutive Activation of FoxO1 Inhibits Differentiation of Human Pancreatic Progenitor Cells

**Figure 5:**
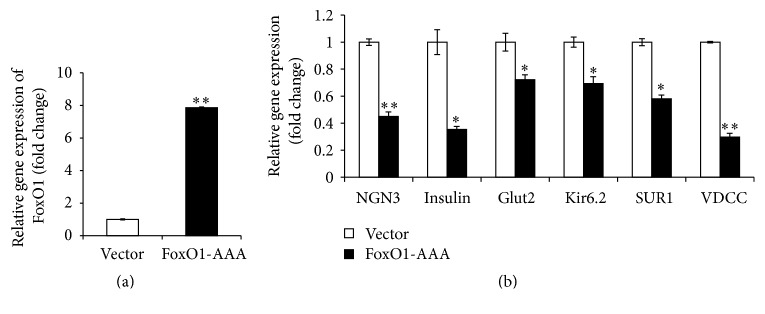
Constitutive activation of FoxO1 results in decrease of *β* cell markers. (a) qRT-PCR analysis of FoxO1 mRNA levels in human pancreatic progenitor cells transfected with retrovirus expressing either vector or constitutively active FoxO1 (FoxO1*-*AAA). Data were normalized against the level of GAPDH and expressed as fold change relative to vector (low panel). Values are means ± SEM of 4 independent experiments per each group. ^*∗∗*^*p* < 0.01. (b) qRT-PCR analysis of *β* cell markers in human pancreatic progenitor cells transfected with retrovirus expressing either vector or constitutively active FoxO1 (FoxO1*-*AAA). Data were expressed as fold change relative to vector and are means ± SEM of 3 to 5 independent experiments per each group. *∗* or *∗∗*, *p* < 0.05 or 0.01.

To confirm the importance of FoxO1 for cell differentiation, we employed gain-of-function approach by transfection of FoxO1-AAA overexpression plasmid (residues Thr24, Ser256, and Ser319 are mutated to Ala) in human fetal pancreatic progenitor cells. In this case, activated FoxO1 would be constantly expressed in the cells. Quantitative analysis revealed that FoxO1-AAA transfected cells exhibited ~8-fold (±0.53; *p* < 0.01) increase of FoxO1 mRNA level, as compared to cells transfected with vector control (Figure 5(a)). Next, we examined the effect of FoxO1 overexpression on cell differentiation in human fetal pancreatic progenitor cells. As shown in Figure 5(b), overexpression of FoxO1 in human pancreatic progenitor cells resulted in ~30% to ~70% (*p* < 0.05 or 0.01) reduction of mRNA levels of Ngn3, insulin, Glut2, Kir6.2, SUR1, and VDCC, respectively (Figure 5(b)). Thus, FoxO1 played a negative role in cell differentiation of human fetal pancreatic progenitor cells.

## 4. Discussion

It has been established that deficient of *β* cell mass plays a vital role in pathogenic process of type 2 diabetes mellitus. Islet transplantation has been suggested as an effective therapeutic strategy to replenish *β* cell mass in both diabetic animals and subjects [15]. Unfortunately, its application is limited by the shortage of available islet supplies. Human fetal pancreatic progenitor cells could be a potential good source of insulin producing cells, as it has a better self-renewal capacity and readily differentiates into insulin producing cells [16]. Our colleagues reported previously that human fetal pancreatic progenitor cells were readily induced into insulin producing cells with higher insulin content and glucose responsiveness, upon in vitro expanded and differentiated in medium for 4 weeks [3]. However, the molecular of control differentiation is still not known. In this study, we got same differentiated human fetal pancreatic progenitor cells at the 10th gestational week by using the similar approach of induction as reported by Zhang et al. [3]. To analyze the role of FoxO1 in the early stage (8–12 weeks) in human fetal pancreatic development [17], we induced the human fetal pancreatic progenitor cells in vitro for 1 week in our study, which correspond to the 12 week fetal development. Notably, we demonstrate that the transcription factor FoxO1 is present in the human fetal pancreatic progenitor cells and acts as a repressor for cell differentiation during the early fetal pancreatic development.

As a transcription factor, the forkhead transcription factor FoxO1 is known to involve in various biological process, owing to its ability to bind to conserved DNA sequence 5′-TTGTTTAC-3′ [18], thus transcriptionally activating or inhibiting a series of downstream targets. It has been found that FoxO1 preferentially expresses in the adult pancreatic *β* cells [6] and plays a critical role in *β* cell growth [5]. Our present study suggested that FoxO1 was also expressed in human fetal pancreatic progenitor cells (Figure 2), which is in agreement with the report by Al-Masri et al. [17], who found that FoxO1 were widely produced during human fetal endocrine pancreatic development. Thus FoxO1 may be implicated in regulation of *β* cell differentiation in human fetal pancreatic progenitor cells. Three pieces of evidence corroborate this notion. First, FoxO1 was abundantly expressed in human fetal pancreatic progenitor cells at the beginning of induction, whereas FoxO1 level decreased throughout 7-day induction (Figures 2(a) and 2(b)). Second, knockdown (Figure 3) or inhibition (Figure 4) of FoxO1 resulted in significant increase of Ngn3, a critical transcription factor in controlling *β* cell differentiation. Consistently, FoxO1 was found to colocalize with the transcription factor Ngn3 during human fetal endocrine pancreatic development [17]. Increased expression of Ngn3 is accompanied by significant increase of insulin, Glut2, Kir6.2, SUR1, and VDCC, which are essential for mature and function of *β* cells. Third, transfection of human fetal pancreatic progenitor cells with constitutive active FoxO1 resulted in reduced levels of Ngn3, insulin, Glut2, Kir6.2, SUR1, and VDCC (Figure 5).

## 5. Conclusion

In summary, our results indicate the expression and potential function of FoxO1 in the development of human fetal pancreatic progenitor cells. Its inhibitory effects on transcription factors critical for *β* cell differentiation suggest that FoxO1 could be a molecular target for generating insulin producing cells.
